# mSRFR: a machine learning model using microalgal signature features for ncRNA classification

**DOI:** 10.1186/s13040-022-00291-0

**Published:** 2022-03-21

**Authors:** Songtham Anuntakarun, Supatcha Lertampaiporn, Teeraphan Laomettachit, Warin Wattanapornprom, Marasri Ruengjitchatchawalya

**Affiliations:** 1 Bioinformatics and Systems Biology Program, School of Bioresources and Technology, King Mongkut’s University of Technology Thonburi (KMUTT), Bangkok, 10150 Thailand; 2 School of Information Technology, KMUTT, Bang Mod, Thung Khru, Bangkok, 10140 Thailand; 3 Biochemical Engineering and Systems Biology Research Group, National Center for Genetic Engineering and Biotechnology (BIOTEC), National Science and Technology Development Agency at King Mongkut’s University of Technology Thonburi, Bang Khun Thian, Bangkok, 10150 Thailand; 4 Department of Mathematics, Faculty of Science, KMUTT, Bangkok, 10140 Thailand; 5 Biotechnology program, School of Bioresources and Technology, KMUTT, Bang Khun Thian, Bangkok, 10150 Thailand; 6 Algal Biotechnology Research Group, Pilot Plant Development and Training Institute (PDTI), KMUTT, Bang Khun Thian, Bangkok, 10150 Thailand

**Keywords:** Microalgae, Machine learning, Non-coding RNAs, Random Forest, Signature feature, SMOTE

## Abstract

This work presents mSRFR (microalgae SMOTE Random Forest Relief model), a classification tool for noncoding RNAs (ncRNAs) in microalgae, including green algae, diatoms, golden algae, and cyanobacteria. First, the SMOTE technique was applied to address the challenge of imbalanced data due to the different numbers of microalgae ncRNAs from different species in the EBI RNA-central database. Then the top 20 significant features from a total of 106 features, including sequence-based, secondary structure, base-pair, and triplet sequence-structure features, were selected using the Relief feature selection method. Next, ten-fold cross-validation was applied to choose a classifier algorithm with the highest performance among Support Vector Machine, Random Forest, Decision Tree, Naïve Bayes, K-nearest Neighbor, and Neural Network, based on the receiver operating characteristic (ROC) area. The results showed that the Random Forest classifier achieved the highest ROC area of 0.992. Then, the Random Forest algorithm was selected and compared with other tools, including RNAcon, CPC, CPC2, CNCI, and CPPred. Our model achieved a high accuracy of about 97% and a low false-positive rate of about 2% in predicting the test dataset of microalgae. Furthermore, the top features from Relief revealed that the %GA dinucleotide is a signature feature of microalgal ncRNAs when compared to *Escherichia coli, Saccharomyces cerevisiae, Arabidopsis thaliana,* and *Homo sapiens.*

## Introduction

Microalgae are a large and diverse group of organisms, with eukaryotic and prokaryotic species found in freshwater, marine, and terrestrial habitats [1–3]. They possess a broad range of biochemical compounds that potentially impact public health, the economy, foods, pharmaceuticals, medicine, bioenergy, environment, and waste treatment [4–7]. Research has sought to understand the mechanisms of beneficial compound production and ways to apply and commercialize them by exploring gene manipulation and regulation, metabolic pathways, omics, and several advanced technologies [8]. Many publications have reported that noncoding RNAs (ncRNAs) play an important role in regulating gene expression, including mRNA destruction, inhibition of translation, post-transcriptional regulation, and control of chromosome dynamics [9–11]. Moreover, many ncRNAs can be found in various organisms, such as mammals, plants, bacteria, and viruses. A system of RNA interference in the post-transcriptional modification process was first found in unicellular green algae [12]. There are many reports about ncRNAs and their functions in plants [13]. However, identifying ncRNAs from laboratory experiments can be time-consuming and costly. Over the last decade, machine learning algorithms have been used in many research fields and for the identification of ncRNAs. In addition, they have facilitated the comparisons of ncRNAs and the identification of homologous structures in databases using BLAST [14]. These conventional approaches rely on the similarity of sequences and only allow for sequences with a high percentage of identity and coverage with previously reported ncRNAs. In contrast, supervised machine learning integrates many informative features and data learning to create models for the identification of candidate ncRNAs. A number of ncRNA identification tools based on computational prediction are available [15–19]. Nevertheless, these tools were not designed for a specific group of microalgae and might not have discriminative features for microalgae ncRNA identification.

Presently, there are many transcriptome data from next-generation sequencing for microalgae. In addition, RNA sequencing has enabled the discovery of many coding transcripts and ncRNAs in particular conditions. This research intends to develop an ncRNA identification tool that can discriminate ncRNAs from partial coding or coding sequences (CDS) in microalgae, including diatoms, golden algae, green algae, and cyanobacteria, using classifier algorithms in a machine learning approach. We applied the synthetic minority oversampling technique (SMOTE), which uses the *k*-nearest neighbor to synthesize new data from a minority class, to address the challenge of data imbalance due to the different numbers of samples in each data class. The significant features for ncRNA identification were selected to improve performance, increase accuracy and reduce false positives. The proposed tool will facilitate discovering and classifying novel ncRNAs from the huge amounts of data from transcriptome studies enabled by next-generation sequencing technologies.

## Material and methods

### Datasets

Noncoding RNAs (ncRNAs) and partial/coding sequences of microalgae were retrieved from the European Bioinformatics Institute (EBI) RNA-central database (ftp://ftp.ebi.ac.uk/pub/databases/RNAcentral) and the NCBI database, respectively. The microalgae included diatoms, golden algae, green algae, and cyanobacteria. In addition, we removed partial or coding sequences that are longer than 800 nt according to the size limitation of the secondary structure folding tool. The retrieved sequences were randomly divided into two parts; 80% of the data was used as a training dataset and the remaining 20% as a test dataset. The data was, unfortunately, highly unbalanced. For example, in the training dataset, there was a much higher number of cyanobacterial ncRNAs (13,116 sequences) compared to those from eukaryotes (3375 sequences). To address the problem, we applied random-sampling and over-sampling techniques to the original training dataset to create a balanced training dataset.

Since the numbers of ncRNA and CDS sequences from golden algae and CDS from diatoms were much fewer than 1125, we applied the SMOTE technique to the datasets. SMOTE was implemented in Weka [20] using the default parameter. It works by recreating new instances from two or more neighbor instances of the same class in the feature space. On the other hand, the over-represented samples, such as the 13,116 prokaryotic ncRNAs and 5448 prokaryotic CDS, were each reduced to 3375 sequences by random selection. The balanced dataset includes 3375 cyanobacterial ncRNAs, 3375 eukaryotic ncRNAs (1125 sequences from each of the three eukaryotic species), 3375 cyanobacterial CDS sequences, and 3375 eukaryotic CDS sequences (1125 sequences from each of the three eukaryotic species) (Table 1).

**Table 1:** Datasets of ncRNAs and CDS of microalgae

Group of microalgae	Types of sequences	Training dataset	Training dataset after balancing	Test dataset
Diatom	ncRNAs	1234	1125^a^	308
	CDS	356	1125*	88
Golden algae	ncRNAs	168	1125*	41
	CDS	60	1125*	15
Green algae	ncRNAs	1973	1125^a^	493
	CDS	6818	1125^a^	1704
Cyanobacteria	ncRNAs	13,116	3375^a^	3280
	CDS	5448	3375^a^	1363

^a^Data generated by random selection; * Data generated by SMOTE

### Features

Four categories of the total 106 features, namely sequence-based, secondary structure, base-pair, and triplet sequence-structure features (Table 1s), were collected from the HLRF tool [21, 22]. Other features in HLRF such as the tetra-nucleotide (4-mer) and the group of structural robustness features, were removed because the performance of 4-mer features was similar to that of 3-mer and 2-mer features combined with secondary structure features [14] and the group of structural robustness features that are suitable for the identification of precursor miRNAs, respectively. Descriptions of the features are provided in Supplementary Material 1s – 4s.

### Feature selection

Student’s t-test, Wilcoxon rank-sum test, Information gain [23], OneR [24], and Relief [25] were used to select the top 20 features that potentially discriminate between ncRNAs and coding sequences of the four groups of microalgae. For Student's t-test and Wilcoxon rank-sum test, the p-values according to the statistical testing were utilized to rank features. Information gain ranks features using the entropy score. OneR uses a rule-based classification algorithm to calculate feature importance. Lastly, the Relief feature selection technique ranks features using the distance to the nearest neighbors.

### Performance measurement

We used the accuracy (ACC), sensitivity (Sn), specificity (Sp), and false-positive rate (FPR) to evaluate the performance of the classifier model prediction. The performance indices were calculated as ACC = (TP + TN)/(TP + TN + FP + FN), Sn = TP/(TP + FN), Sp = TN/(TN + FP) and FPR = FP/(TN + FP), where TP, TN, FP, and FN are the numbers of true positives, true negatives, false positives, and false negatives, respectively. For algorithm selection, six classifier algorithms, namely Naïve Bayes (NB), Random Forest (RF), Neural Networks (NN), K-nearest neighbor (KNN), Decision Tree (DT), and Support Vector Machine (SVM), were evaluated. They were trained, tested, and compared using default parameters on the open-source data mining software Weka [20]. For the Random Forest model, in particular, the number of trees was set to 100, and the maximum depth of the tree was set to unlimited.

### Model building

**Fig. 1:**
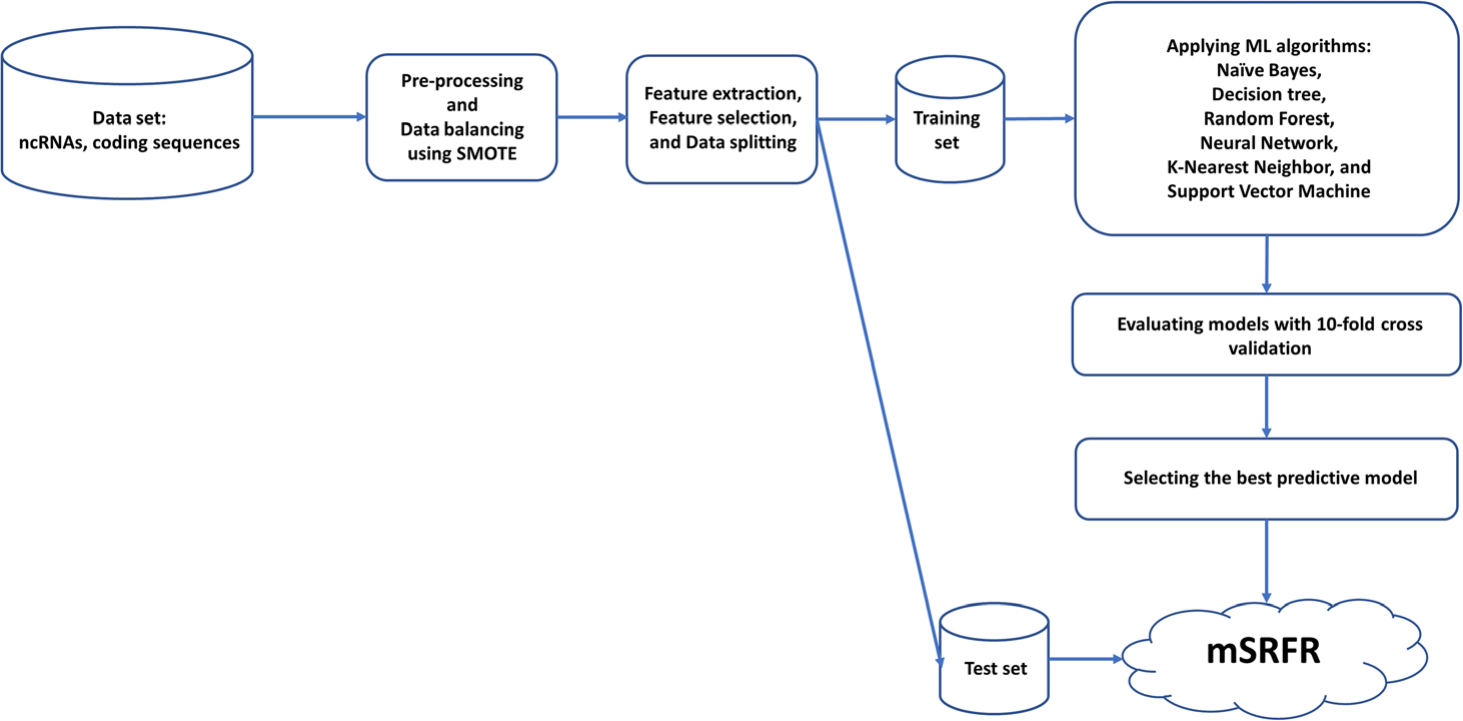
The overall workflow of this work. Feature selection was performed, and machine learning models were applied. Finally, the models were evaluated by 10-fold cross-validation, and the best model was tested with a test set

In total, samples in the training dataset include 6750 ncRNAs (positive dataset) and 6750 partial coding sequences (negative dataset) from eukaryotic and prokaryotic algae. A total of 106 features were extracted from the sample data. Then the best classifier algorithm among NB, DT, RF, NN, KNN, and SVM was chosen based on their performance in classifying the positive and negative datasets using all 106 features. Subsequently, the selected classifier algorithm was used to classify the positive and negative datasets again, but with only the top features extracted by the five feature selection methods (Student’s t-test, Wilcoxon rank-sum test, Information gain, OneR, and Relief). Top features that yielded the highest performance were used in the next step for 10-fold cross-validation with the six classifier algorithms. Finally, the best-performing classifier algorithm was selected as the final model and was further evaluated with the test dataset (20% of all data shown in Table 1). The overall workflow of the present work is shown in Fig. 1.

### Identification of unique features of ncRNAs of microalgae

The unique and common top features extracted by the feature selection methods were analyzed and illustrated with a Venn diagram.

## Results

### Feature selection model for microalgal ncRNA classification

Firstly, a suitable classifier was chosen by comparing the performance of six different classifier algorithms, NB, DT, RF, NN, KNN, and SVM, using all 106 features. Results of the 10-fold cross-validation of each classifier algorithm are shown in Table 2s. RF gave the highest ROC area, while NN had slightly higher accuracy, sensitivity, specificity, and a lower false-positive rate. Nevertheless, RF is less computationally intensive regarding training and less prone to overfitting compared to NN [26], and its built-in feature importance graphical plots are easier to interprete and comprehend [27]. Moreover, RF has been widely used in many classification tools for ncRNAs prediction [21, 22]; therefore, we selected it as a classifier to compare the performance of a set of top features from different feature selection methods in the following step.

Various feature selection methods, namely Information gain, OneR, Relief, t-test, and Wilcoxon rank-sum test, were used to identify features that can potentially distinguish microalgae ncRNA sequences from coding sequences. The top features identified by each feature selection method are shown in Table 3s. They comprise 5 sequence-based features, 12 secondary structure features, and 3 base-pair features by Information gain, 15 sequence-based features, 3 secondary structure features, and 2 base-pair features by OneR, 13 sequence-based features, 4 secondary structure features, 2 base-pair features, and 1 triplet sequence-structure feature by Relief, 4 sequence-based features, 14 secondary structure features, 10 base-pair features and 4 triplet sequence-structure features by Student’s t-test, and 4 sequence-based features, 16 secondary structure features, 13 base-pair features, and 5 triplet sequence-structure features by Wilcoxon rank-sum test. Results of the 10-fold cross-validation of the top features from each feature selection method using the Random Forest algorithm are shown in Table 2. The Relief method yielded the best performance in terms of the accuracy, sensitivity, specificity, and false-positive rate. This may be because Relief employs filtering algorithms, and weights computed for individual features can guide downstream machine learning. In addition, this technique does not remove feature correlations or feature redundancies [28].

**Table 2:** Performance of different feature selection methods with Random Forest algorithms using 10-fold cross validation

Feature selection methods	Performance measurement				
	ACC (%)	Sn (%)	Sp (%)	FPR	ROC area
Infogain	98.7	98.7	98.7	0.013	0.999
OneR	98.9	98.9	98.9	0.011	0.999
Relief	98.9	99.0	99.0	0.01	0.999
t-test	98.3	97.7	98.9	0.011	0.999
Wilcoxon	98.6	98.4	98.9	0.011	0.999

We used a Venn diagram to visualize the top features (Fig. 1s) and compare the feature selection methods (Table 4 s). Only four features, namely TmL, Prob, CM, and GA, were commonly selected by all methods. To prove the importance of these features for the identification of ncRNAs in microalgae, we constructed a Random Forest model using only the four common top features (F4model) and then compared its performance to that of the original model (F20model), which was based on the 20 features (Table 5 s). The F4 model achieved a 95% accuracy compared to 97% for the F20 model, indicating that the four features contributed majorly to the identification of ncRNAs in microalgae. In addition, three of the features (TmL, Prob, CM) are secondary structure features, a probable indication of the relevance of secondary structure features. For instance, the structural entropy-derived dS feature, also found in the top 20 significant features, represents the fold stability of RNA sequences and can be used to classify precursor miRNAs from pseudo precursor miRNA sequences [29]. In addition, the TmL feature, which represents the melting energy of structure normalized by the sequence length, has been reported to discriminate between ncRNAs and coding RNAs [30]. In fact, secondary structure features have been used in a wide range of ncRNA identification tools such as HeteroMirPred [21] and HLRF [22], and to identify mature miRNAs from precursor miRNAs [31]. Therefore, the top 20 features selected by the Relief method (F20 model) were used for further analysis.

#### Unique ncRNA features of microalgae

**Fig. 2:**
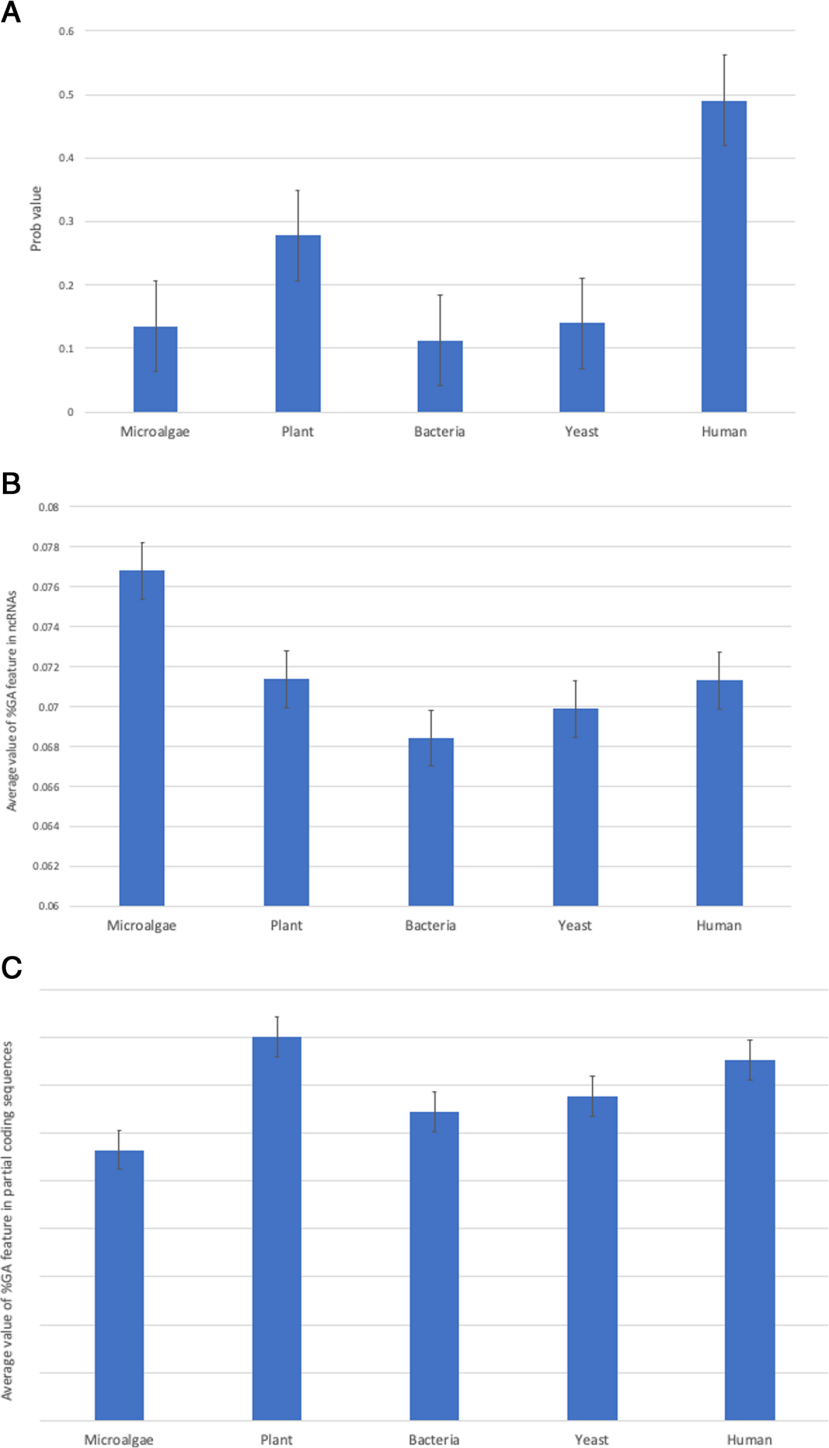
Comparison of the features from microalgae to bacteria, yeast, plants and humans: average Prob values (**A**), average %GA values in ncRNAs (**B**), and average %GA values in partial coding sequences (**C**)

**Fig. 3:**
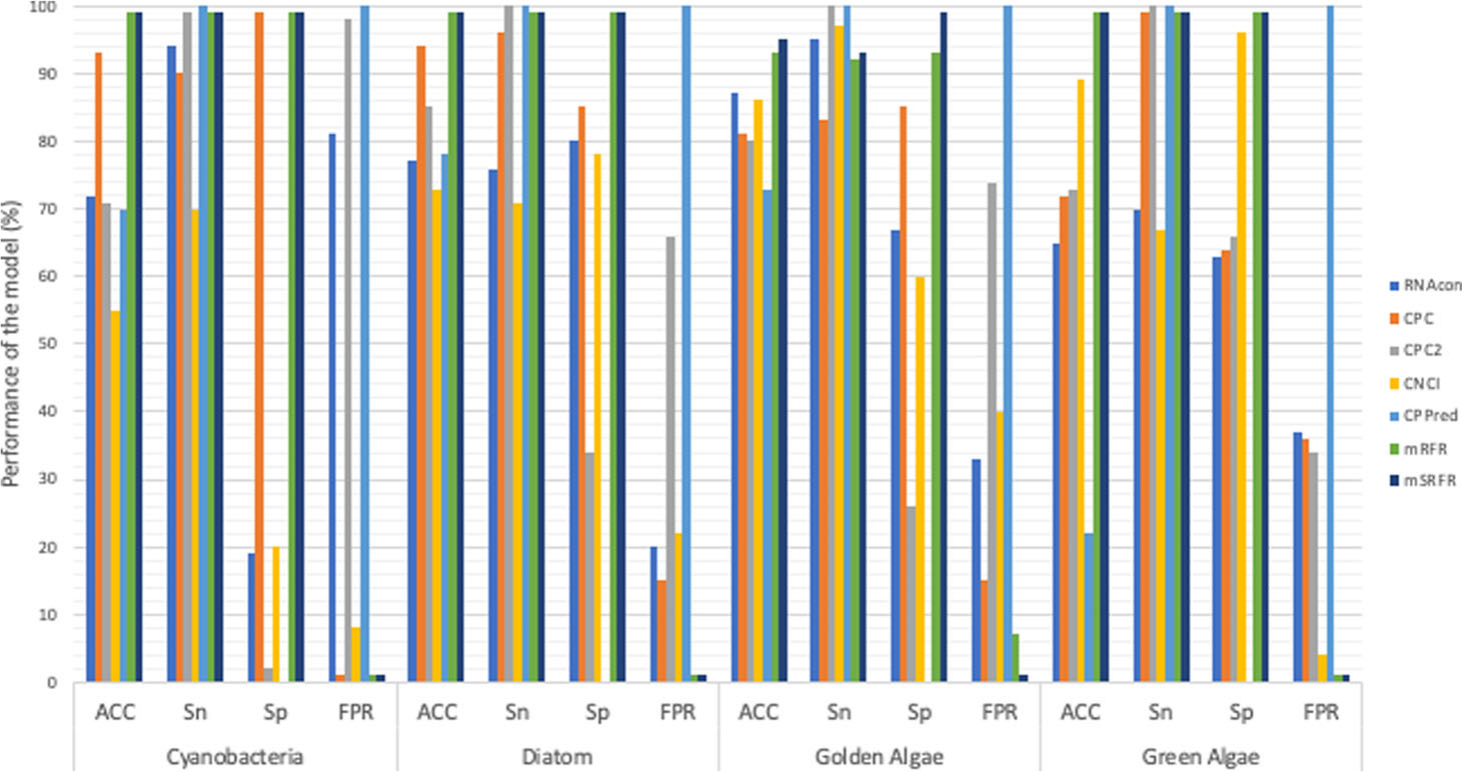
Performance comparison of our model, with or without applying the SMOTE technique and other tools (RNAcon, CPC, CPC2, CNCI, and CPPred) to discriminate between ncRNAs and CDS in the test dataset

We compared the microalgal ncRNA top features with those of other organisms, including bacteria, yeast, plants, and humans. Venn diagrams illustrating the top 20 features selected by the different feature selection methods, including Information gain, OneR, Relief, t-test, and Wilcoxon rank-sum test, are presented in Figs. 2s–5s. The top features that were commonly selected by all methods are as follows: TmL, pairprob7, AG, CM, and GG for bacteria (*Escherichia coli*); dF and TmL for yeast (*Saccharomyces cerevisiae*); div, pairprob8, pairprob4, dH, pairprob7, TmL, pairprob9, CM, efe, dS, pairprob3, mfe and Tm/Loop for plants (*Arabidopsis thaliana*); and dH, TmL, pairprob9, pairprob2, diff, Tm/Loop, dS and mfe5 for humans (*Homo sapiens*). Interestingly, GA and Prob features were unique to microalgae, i.e., they were not among the top features for bacteria, yeast, plants, or humans.

#### Comparison of GA and Prob values of ncRNAs from microalgae to those from other organisms

We compared the two unique features of the microalgal ncRNAs, namely the average GA and Prob values, to the values of ncRNAs from other organisms (Fig. 2A). The Prob feature value of ncRNAs from microalgae was significantly smaller than that of plant and human ncRNAs but was comparable to that of yeast and bacterial ncRNAs. Moreover, the %GA dinucleotide (GA) in microalgal ncRNAs was significantly higher (*p*-value < 0.01) than in the bacterial, yeast, plant, and human ncRNAs (Fig. 2B), whereas the %GA dinucleotide of coding sequences in microalgae was significantly less (*p*-value < 0.01) in comparison (Fig. 2C). From these results, it can be concluded that the %GA dinucleotide is potentially a signature feature of microalgal ncRNAs considering its abundance.

### Comparison of performance of different classifier algorithms

Results of the performance evaluation of the six classifier algorithms, namely Decision Tree (DT), K-nearest neighbor (KNN), Naïve Bayes (NB), Neural Network (NN), Random Forest (RF), and Support Vector Machine (SVM), using 10-fold cross-validation with the top 20 features selected by the Relief method are shown in Table 6 s. The classifier algorithms mostly showed comparable performance. However, the Random Forest algorithm had the highest accuracy, specificity, ROC area, and the lowest false positive rate and was thus chosen, as the false positive rate is particularly important for identifying microalgal ncRNAs. Random Forest, as an ensemble of multiple decision trees, could be useful for the identification of noncoding RNAs in microalgae, just as it has been used for a broad range of organisms [21].

### Benchmarking of our mSRFR model performance with other tools

We evaluated the performance of the trained microalgal Random Forest model using the top 20 features from the Relief feature selection method (mSRFR model) to identify ncRNAs from the test dataset. We benchmarked our model against other classification tools, namely RNAcon [14], CPC [15], CPC2 [16], CNCI [17], CPPred [18], and a Random Forest model using the top 20 features from the Relief feature selection but without applying the SMOTE technique to balance the dataset (mRFR model). As shown in Table 3 and Fig. 3, the results are divided into four groups: cyanobacteria, diatoms, gold algae, and green algae. The accuracy of our mRFR was high and similar to mSRFR. However, for golden algae, the accuracy of mSRFR was higher by around 2%. In addition, mSRFR showed the lowest false positive rate when compared with the other tools.

**Table 3:** Performance comparison of our mSRFR model to others (RNAcon, CPC, CPC2, CNCI, and CPPred) used in discriminating ncRNAs and CDS of microalgae

		RNAcon	CPC	CPC2	CNCI	CPPred	mRFR	mSRFR
Cyanobacteria	ACC (%)	72	93	71	55	70	99	99
	Sn (%)	94	90	99	70	100	99	99
	Sp (%)	19	99	2	20	0	99	99
	FPR	0.81	0.01	0.98	0.8	1	0.01	0.01
Diatom	ACC (%)	77	94	85	73	78	99	99
	Sn (%)	76	96	100	71	100	99	99
	Sp (%)	80	85	34	78	0	99	99
	FPR	0.20	0.15	0.66	0.22	1	0.01	0.01
Golden Algae	ACC (%)	87	81	80	86	73	93	95
	Sn (%)	95	83	100	97	100	92	93
	Sp (%)	67	85	26	60	0	93	99
	FPR	0.33	0.15	0.74	0.40	1	0.07	0.01
Green Algae	ACC (%)	65	72	73	89	22	99	99
	Sn (%)	70	99	100	67	100	99	99
	Sp (%)	63	64	66	96	0	99	99
	FPR	0.37	0.36	0.34	0.04	1	0.01	0.01

## Discussion

To the best of our knowledge, this tool is the first ncRNAs classification tool trained specifically for microalgae, the most promising biofuel candidates rich in high-value bioactive compounds. Identifying ncRNAs in microalgae might help improve our understanding of their regulatory functions in gene manipulation. Moreover, ncRNA investigation in microalgae may provide new insights into metabolic regulation and engineering for product improvement.

The main contributions are threefold:

First, we developed an ncRNA identification tool for microalgae-based on a Random Forest model. Random Forest, an ensemble machine learning method, has been used for a broad range of organisms for its robustness and minimal overfitting problems.

Second, to address the problem of imbalanced datasets of different groups of microalgae, for example, where the numbers of both ncRNAs and coding sequences from golden algae were far fewer than those of the other groups, we applied the SMOTE technique to balance the datasets. Interestingly, the SMOTE (mSRFR) model performed better than the model without SMOTE (mRFR) in discriminating ncRNAs from CDS in golden algae, suggesting that the SMOTE model can handle imbalanced datasets better.

Third, a group of features specific to ncRNAs in microalgae were identified. Moreover, our study also revealed four features (TmL, Prob, CM, and GA) that contributed majorly to the performance of the model. The RF classifier model utilizing only these four features, instead of all top 20 features, achieved a reasonably high accuracy of 95%. Our analysis further shows that of the four features, Prob and GA are unique features in discriminating ncRNAs from coding sequences in microalgae. They were not among the top features, by any of the methods, for the bacterial, yeast, plant, or human ncRNAs discrimination. We propose the GA dinucleotide as one of the microalgal ncRNAs signature features, given its higher abundance in microalgal ncRNAs compared to other species. Conversely, the coding sequences of microalgae contained fewer GA dinucleotides in comparison to those of the other organisms. The GA dinucleotide or GA motif is highly conserved in T box and S box sequences, which are transcription termination control systems. The T box functions in regulating amino acid transporter genes, aminoacyl-tRNA synthetase, and amino acid biosynthesis, while the S box is related to methionine synthesis [32]. The high GA dinucleotide composition of microalgal ncRNAs could be relevant for transcription termination control systems as microalgal photosynthesis is highly regulated on the level of transcriptional control [33].

## Conclusions

In this study, mSRFR (m for microalgae; S for SMOTE; RF for Random Forest; R for the Relief method) was used for ncRNA identification in microalgae-based on a Random Forest model using the top 20 features from a total of 106 features selected by the Relief feature selection method. The tool achieved a high accuracy of about 97% and a low false-positive rate of 2% in discriminating microalgal ncRNAs from coding sequences in a test dataset containing 7292 sequences. Currently, next-generation sequencing technologies, such as RNA-seq, have become popular for the study of gene expression in many organisms. For future research, we aim to extend our tool to support input formats of raw reads from next-generation sequencing data, such as fastq, and to develop a web-based computational pipeline that will enable users to identify potential ncRNAs in microalgae from next-generation sequencing data.

## Data Availability

mSRFR: A machine learning model using microalgal signature features for ncRNA classification is an open-source collaborative initiative available in the GitHub repository (https://github.com/anun001/smRFR-smote-microalgae-Random-Forest-Relief-model).
